# Supplementary figures and images for: Construction of a high-density genetic map using specific-locus amplified fragment sequencing and quantitative trait loci analysis for tillering related traits in Psathyrostachys juncea perennial grass
Source: PeerJ. 2024 Nov 6;12:e18409. doi: 10.7717/peerj.18409 (PMC11549907; doi:10.7717/peerj.18409)

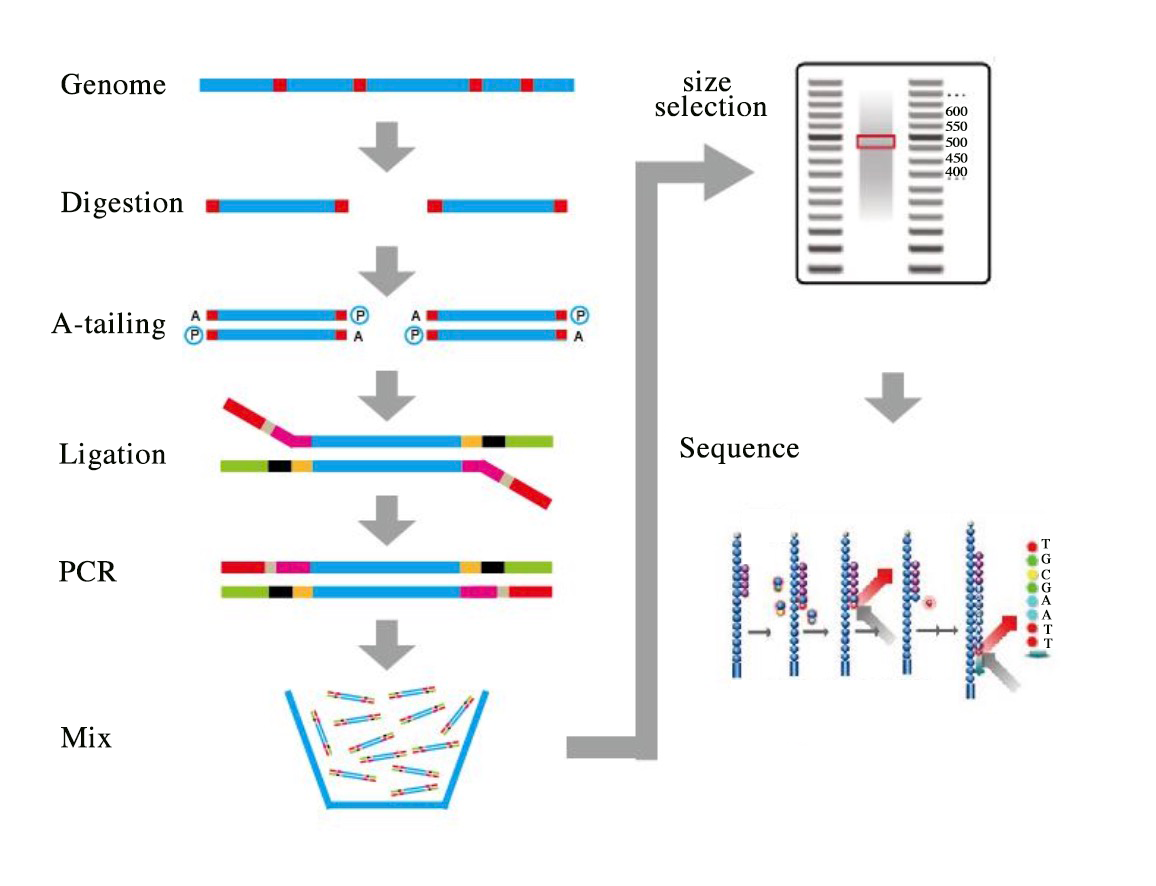

Supplement: Supplemental Information 1 [file peerj-12-18409-s001.png]
